# Inflammatory Determinants of Differential Tuberculosis Risk in Pre-Adolescent Children and Young Adults

**DOI:** 10.3389/fimmu.2021.639965

**Published:** 2021-02-25

**Authors:** Richard Baguma, Stanley Kimbung Mbandi, Miguel J. Rodo, Mzwandile Erasmus, Jonathan Day, Lebohang Makhethe, Marwou de Kock, Michele van Rooyen, Lynnett Stone, Nicole Bilek, Marcia Steyn, Hadn Africa, Fatoumatta Darboe, Novel N. Chegou, Gerard Tromp, Gerhard Walzl, Mark Hatherill, Adam Penn-Nicholson, Thomas J. Scriba

**Affiliations:** ^1^ South African Tuberculosis Vaccine Initiative (SATVI), Department of Pathology, Institute of Infectious Disease and Molecular Medicine and Division of Immunology, University of Cape Town, Cape Town, South Africa; ^2^ DST-NRF Centre of Excellence for Biomedical Tuberculosis Research, South African Medical Research Council Centre for Tuberculosis Research, Division of Molecular Biology and Human Genetics, Department of Biomedical Sciences, Faculty of Medicine and Health Sciences, Stellenbosch University, Cape Town, South Africa

**Keywords:** inflammation, tuberculosis, anti-mycobacterial immunity, age, pediatric

## Abstract

The risk of progression from *Mycobacterium tuberculosis* (*M.tb)* infection to active tuberculosis (TB) disease varies markedly with age. TB disease is significantly less likely in pre-adolescent children above 4 years of age than in very young children or post-pubescent adolescents and young adults. We hypothesized that pro-inflammatory responses to *M.tb* in pre-adolescent children are either less pronounced or more regulated, than in young adults. Inflammatory and antimicrobial mediators, measured by microfluidic RT-qPCR and protein bead arrays, or by analyzing published microarray data from TB patients and controls, were compared in pre-adolescent children and adults. Multivariate analysis revealed that *M.tb*-uninfected 8-year-old children had lower levels of myeloid-associated pro-inflammatory mediators than uninfected 18-year-old young adults. Relative to uninfected children, those with *M.tb*-infection had higher levels of similar myeloid inflammatory responses. These inflammatory mediators were also expressed after *in vitro* stimulation of whole blood from uninfected children with live *M.tb*. Our findings suggest that myeloid inflammation is intrinsically lower in pre-pubescent children than in young adults. The lower or more regulated pro-inflammatory responses may play a role in the lower risk of TB disease in this age group.

## Introduction

Infection with *Mycobacterium tuberculosis (M.tb)* predisposes to pulmonary tuberculosis (TB) disease, which kills more people than any other infectious agent (1). The risk of progression from infection to disease varies markedly with age. Infants and young children are at very high risk of TB disease following infection. However, pre-adolescent children above 4 years of age, in the so-called “Golden Age” or “Wonder Years” (2), are curiously at significantly lower risk of active TB disease compared with post-pubescent adolescents and young adults (3–5). Upon first thought this pattern may be interpreted to follow the prevalence of underlying *M.tb* infection, which increases with age during childhood in settings endemic for TB (6). However, the dramatic increase in TB disease risk during puberty cannot be sufficiently explained by changes in *M.tb* infection incidence, which appears to increase only marginally during the same period (2). The clinical presentation of TB is also different by age. TB typically manifests as mild and/or pauci-bacillary lymph node disease in pre-adolescent children of the Golden Age (3–5). By contrast, post-pubescent adolescents and adults more commonly present with multi-bacillary, “adult-type” pulmonary disease with more pronounced immunopathology, including cavitary disease (2–5). These phenomena have led us (7) and others (2) to surmise that pre-pubescent children have more effective or successful immunity against *M.tb* and that the onset of puberty coincides with or brings about immunological changes that result in less successful control of the infection.

The determinants of effective immune control of *M.tb* in humans are thought to be dependent on a complex collaboration of multiple adaptive and innate immune responses that must be maintained in a manner that balances pro- and anti-inflammatory responses (8, 9). Although intact antigen-specific T cell responses of the Th1 lineage are necessary for host resistance against TB, based on current evidence from humans the magnitude or functional characteristics of such Th1 responses are not associated with clinical outcome of *M.tb* infection [reviewed in (10)]. However, mounting evidence suggests that excessive skewing of the inflammatory milieu towards either a pro-inflammatory or an anti-inflammatory response can lead to a loss of immunological control of *M.tb* and TB disease progression (11). For example, dysregulated TNF responses in mycobacterial infection have been associated with detrimental outcome of TB in zebrafish and humans (12). Furthermore, upregulation of Type I IFN responses during murine influenza infection impairs control of subsequent *M.tb* challenge (13). Elevated Type I/II IFN is also measured as a blood biomarker of incipient or subclinical TB disease in *M.tb-*infected humans, and blood transcriptomic signatures that detect such Type I/II IFN responses can be used to identify individuals at high risk of incident TB (14–17). Conversely, peripheral blood neutrophil count has been inversely associated with risk of TB and this neutrophil-mediated bacterial control was dependent on the neutrophil peptides, cathelicidin LL-37 and lipocalin-2 (18).

The low risk of TB in pre-adolescent children presents an opportunity to study natural resistance and/or characteristics of successful immunity to *M.tb* in humans. We previously characterized frequencies and functions of antigen-specific T cell responses in pre-adolescent children and young adults and observed no differences (7). We also measured mycobacterial growth inhibition in whole blood from pre-adolescent children and young adults as an *in vitro* surrogate of differential immunological control of *M.tb*, but also observed no significant differences between these age groups (7).

Here, we hypothesized that pro-inflammatory responses to *M.tb* in pre-adolescent children are either less pronounced or more regulated, than in young adults.

We compared blood gene expression of antimicrobial effector molecules, pro- and anti-inflammatory and other immune mediators as well as soluble host-derived inflammatory molecules in healthy *M.tb*-infected or uninfected 8-year-old children and 18-year-old young adults. Our analyses reveal that uninfected 8-year-old children had lower levels of myeloid-associated pro-inflammatory mediators than uninfected 18-year-old young adults. Relative to uninfected children, those with *M.tb*-infection had higher levels of similar myeloid inflammatory responses. These inflammatory mediators were also expressed after *in vitro* stimulation of whole blood from uninfected 8-year-old children with live *M.tb*.

## Materials and Methods

### Ethics Statement

Participants were recruited under protocols approved by the University of Cape Town Human Research Ethics Committee. Written informed consent was obtained from adults prior to enrolment. Children provided written informed assent while their legal guardians provided written informed consent prior to enrollment.

### Study Design and Participants

This was a cross-sectional study comprising two cohorts of healthy, HIV-negative participants from the Worcester region in the Western Cape Province of South Africa, a setting with high burden of TB. Individuals with any acute or chronic disease, those taking immunosuppressive medication, with a previous diagnosis of active TB disease or who currently or previously participated in a TB vaccine trial, were excluded. Women who were pregnant or lactating were also excluded from participation. We aimed to enroll young adults aged 18 years and 8-year-old children with equal numbers of *M.tb*-infected and uninfected participants in each age group. *M.tb*-infection status was assessed with the QuantiFERON-TB Gold In-Tube Assay (QFT) (Qiagen), according to the manufacturer’s instructions. HIV infection was diagnosed by rapid HIV-antibody test. Some aspects of these cohorts have been described before (7).

Venous blood was collected from participants directly into QFT tubes, into sodium heparin tubes for whole blood stimulation assays and into Cell Preparation Tubes (CPT) for isolation of peripheral blood mononuclear cells (PBMC).

We also analyzed two whole blood microarray datasets from children and adults with microbiologically-confirmed TB or latent *M.tb* infection (LTBI controls), published previously (19, 20). Datasets were sourced from the NCBI database Gene Expression Omnibus (GEO); analyses are described further below.

### Mycobacterial Strains and Whole Blood Stimulations

Three mycobacterial strains (*M.tb* H37Rv, *M.tb* HN878 and *M.tb* CDC1551), selected to encompass a range of virulence and inflammation-inducing *M.tb* strains, with HN878 known to be more virulent than *M.tb* H37Rv and *M.tb* CDC1551 (21, 22), were separately added to Sarstedt tubes containing 300 µl RPMI at volumes equating to 5 x 10^5^ colony forming units [CFU]/ml inocula. 300 μl fresh heparinized whole blood was added and the tubes were incubated for 12 h at 37°C with slow constant rotation. Bacilli were sedimented at 208 x g for 10 min. The supernatants were aspirated and filtered through a 0.22 μm (pore-size) Costar^®^ Spin-X^®^ centrifuge tube filter (Corning, USA) for 2 min at 14,000 x g before storing at -80°C for later measurement of soluble host marker levels.

### Peripheral Blood Mononuclear Cells Culture

Cryopreserved PBMC were thawed into medium containing DNase (50 IU/ml, Sigma-Aldrich), cell viability assessed, counted and plated out at 1 x 10^6^ cells/well in RPMI 1640 media with 10% human AB serum (Sigma-Aldrich). Following overnight rest at 37°C, 5% CO_2_, PBMC were then lysed and homogenized with 350 µl/well of Buffer RLT Plus (Qiagen, USA) and stored at -80°C for later RNA isolation.

### RNA Extraction, Reverse transcription, and Specific Target/Transcript Amplification

RNA was extracted from PBMC lysates using the RNeasy Plus Micro kit for purification of total RNA from animal cells (Qiagen, Valencia, CA) according to the manufacturer’s instructions. RNA yield and purity were determined using a Nano Drop ND 2000 spectrophotometer (Thermo Scientific, Waltham, MA).

Reverse transcription was performed using 100 ng of purified total RNA in a 20 µl reaction volume containing 1 µl 10mM dNTP (Sigma-Aldrich, USA), 1 µl Oligo(dT)_12-18_ (Sigma-Aldrich, USA), 4 μl 5X First-Strand Buffer (Invitrogen, USA), 2 µl 0.1M DTT (Invitrogen, USA), 0.5 μl RNaseOUT™ (40 U/μl) (Invitrogen, USA), 0.5 µl sterile endotoxin free water (dH_2_0) and 1 µl SuperScript™ III Reverse Transcriptase (200 units) (Invitrogen, USA), according to the manufacturer’s instructions. cDNA was then diluted 1:5 with dH_2_0.

The protocol for microfluidic RT-qPCR has been previously described (16, 23). In order to increase the number of cDNA templates for downstream microfluidic RT-qPCR where the cDNA is dispensed into 96 nanowells, a pre-amplification PCR was performed at 10 μl containing 5 μl of 2X TaqMan PreAmp Master Mix (Thermo Fisher Scientific, USA), 2.5 μl of 0.2X 96-pooled TaqMan Gene Expression (GE) assay (Thermo Fisher Scientific, USA) mix and 2.5 µl of 1:5 diluted cDNA using the following thermal profile: one cycle at 95°C for 10 min, followed by 16 cycles of 95°C for 15 s and 60°C for 4 min. Pre-amplification products were diluted 1:25 with dH_2_0 and stored at -80°C until needed.

Confirmation of successful pre-amplification was done on a Rotor-Gene 6000 RT-PCR

instrument (Corbett Life Science) in a 20 µl reaction volume consisting of 10 µl of 2X TaqMan Universal PCR Master Mix, 1 µl of 20X select TaqMan GE assay and 4 µl of 1:25 diluted pre-amplified cDNA made up to a final volume of 20 µl with dH_2_0. The cycling program consisted of a 2 min incubation at 50°C, a 10 min incubation at 95°C, followed by 40 cycles of 95°C for 15 s and 60°C for 1 min.

### High-Throughput Microfluidic RT-qPCR

We selected a total of 87 genes based on broad relevance to inflammation, immunopathology, antimycobacterial response, type I IFN response, immune regulation as well as risk for TB (see **Table S1** in **Supplementary Material**). Expression levels of these genes were measured using the previously described high-throughput microfluidic 96.96 Dynamic Array and BioMark™ HD instrument (Fluidigm, USA) (24). Specifically, a 5 µl sample mix was prepared for each sample containing 2.5 µl of 2X TaqMan Universal PCR Master Mix (Applied Biosystems, PN 4304437), 0.25 µl of 20X GE Sample Loading Reagent (Fluidigm PN 85000746) and 2.25 µl of pre-amplified cDNA (diluted 1:25). 5 µl of assay mix was prepared with 2.5 µl of each 20X TaqMan GE Assay (Thermo Fisher Scientific, USA) and 2.5 µl of 2X Assay Loading Reagent (Fluidigm PN 85000736). An IFC Controller HX (Fluidigm, USA) was used to prime the Dynamic Array™ (chip) with control line fluid and before loading the sample and assay mixes into their appropriate inlets. The chip was subsequently returned to the IFC Controller HX for loading and mixing. After approximately 60 min, the chip was transferred to the BioMark™ HD instrument for RT-qPCR according to the manufacturer’s protocol.

### Luminex Multiplex Immunoassay

The concentrations of 24 soluble host-derived markers were considered for measurement in supernatants from the 12 h cultures (*see above*) using MILLIPLEX^®^ MAP kits (Millipore, Billerica, MA). These markers were selected for potential relevance as essential role players in the inflammatory processes likely to be affiliated with age-associated differential risk of TB. These markers were analyzed in 15-plex (TNF-α, MIP-1α, MIP-1β, MCP-1, IL-10, IL-1α, IL-1RA, sCD40L, Fractalkine, IFN-γ, IFN-α2, IP-10, IL-4, IL-15 and VEGF), 5-plex (MMP-1, MMP-2, MMP-7, MMP-9, and MMP-10), 1-plex (D-dimer), and 3-plex (CRP, Fibrinogen, and SAP) assays in 1:2, 1:100, 1:100, and 1:20,000 dilutions, respectively. IL-4, IL-15, MMP-7, and MMP-10 were not detectable and were thus omitted from further evaluation. For samples in which marker concentrations were extrapolated and/or were above the Upper Limit of Quantitation (ULOQ) or below the Lower Limit of Quantitation (LLOQ), readings were assigned values calculated as follows:

- <OOR (reading below LLOQ) = {value of lowest standard X dilution factor}
- OOR> (reading above ULOQ) = {highest standard X dilution factor} + 50

Internal controls were included throughout each run. In summary, following pre-wetting of the filter plates with 200 µl of wash buffer, 25 µl of standards, controls and diluted samples were added to each well containing 25 µl of assay buffer. Pre-combined beads of all the 24 individual markers were added to all wells before incubating sealed plates with agitation for 1 h in the dark at room temperature. The plates were washed twice and 25 µl of detection antibody was added, and the sealed plates incubated with agitation for 30 min. Streptavidin-Phycoerythrin (25 μl per well) was added and incubated for 10 min. Plates were washed twice and 150 μl of sheath fluid was added to each well. The beads were re-suspended by agitation for 5 min before running the plates on a Bio Plex 200 instrument (Bio Rad Laboratories, Hercules, CA, USA). Data were acquired and analyzed using the Bio Plex Manager 6.1 Software (Bio Rad).

### Statistical Analysis

All appropriate descriptive statistics applied to RT-qPCR and Luminex data were performed using R for statistical computing (25) or PRISM (GraphPad Software v8, San Diego, Calif.).

Previously published microarray data from children aged 4–12 years and adults with microbiologically-confirmed TB or LTBI controls were processed in the same facility and run on Illumina HT12 beadarrays: GSE39940 and GSE37250 (19, 20). Each array was separately renormalized *via* normal-exponential background correction followed by quantile normalization and log_2_-transformation. In order to reduce the potential influence of batch effects, we applied surrogate variable analysis implemented in the R package sva (26). The demographic and phenotypic annotation reported by the original authors were used to subset the samples into HIV negative children aged 4 to 12 years and adults, sensitized to *M.tb* (*M.tb* infected controls) and those with microbiologically-confirmed TB disease (see **Table S2** in **Supplementary Material**). Discrimination between TB and *M.tb* infected controls by the Sweeney3 and RISK6 signatures was done using signature scores generated as previously described (16, 27, 28). For the Sweeney3 signature (27), scores were computed for each sample by subtracting the geometric mean of GBP5 and DUSP3 from the geometric mean of KLF2 in R as implemented in MetaIntegrator package (29) on the gene-level expression data. Changes in cell subset proportions were performed with immunoStates (30).

Linear models were fitted with R/Bioconductor *limma* package (31) to extract the age effect on genes differentially expressed in TB disease between pre-adolescents and adults. Genes were considered differentially expressed with false discovery rate (FDR)-adjusted at 1% with at least an absolute log_2_-fold change of 0.5. Genes were tested for enrichment using blood transcriptional modules (BTMs) developed by Chaussabel and co-workers (32) within the tmod R package (33) and the CERNO statistical test was applied on the genes ordered by minimum significant difference that uses combination of effect size and statistical significance.

Microfluidic RT-qPCR data were analyzed using the BioMark™ Real-Time PCR Analysis Software in the BioMark™ HD instrument (Fluidigm, CA) to obtain threshold cycle (Ct) values. Analysis settings were as follows: amplification curve quality threshold was set to 0.65, baseline correction to linear (derivative) and Ct threshold method to auto (global). For ease of interpretation, Ct values were transformed to Et values (40 − Ct), because a higher Et value indicates higher mRNA levels. Results were presented as changes in relative mRNA transcript expression normalized with the geometric mean of the Et values of the reference genes (34). TB risk signature scores were calculated as previously described (28). P values were adjusted to account for multiple comparisons. The method for each analysis is indicated in the *Figure Legends*.

Comparison of soluble host-derived markers was assessed using the non-parametric Mann-Whitney U and Wilcoxon matched-pairs signed-rank tests to determine differences between groups (18 compared to 8 year olds and *M.tb*-infected (QFT+) compared to uninfected (QFT-) individuals) and within groups (*M.tb* H37Rv, *M.tb* HN878 and *M.tb* CDC1551-stimulated samples) respectively. Unstimulated values were considered as background and subtracted from the *M.tb* stimulated values before further analysis. Soluble host-derived marker median concentration point estimates and 95% confidence intervals of the differences between groups were calculated using the rank inversion method and bootstrapping 2000X using the quantreg package in R (35). To visualize the groups of participants and groups of markers with similar expression profiles, unsupervised hierarchical clustering (i.e. heatmaps) was performed using factoextra (36), FactoMineR (37) and ComplexHeatmap (38) packages, respectively in R (25). P values were adjusted to account for multiple comparisons. The method for each analysis is indicated in the *Figure Legends*.

Data from all enrolled participants including microfluidic RT-qPCR and soluble host-derived markers are available on FigShare (https://doi.org/10.25375/uct.13351466.v1).

## Results

### Study Participants

In total, 117 healthy participants were enrolled into the two cohorts of this study: 53 children aged 8 years, and 64 young adults aged 18 years. Participants in each age group were approximately equally stratified according to *M.tb* infection status by QFT, which shows excellent agreement with tuberculin skin test (TST) in this setting (39, 40). The demographic characteristics of the study participants are summarized in **Table 1**.

**Table 1:** Summary of demographic characteristics of study participants.

	Children		Young adults	
	QFT+	QFT-	QFT+	QFT-
**Participants, n**	25	28	39	25
**Female, n (%)**	11 (44)	15 (54)	22 (56)	13 (52)
**Ethnicity, n (%)**				
** Black African**	0 (0)	3 (11)	18 (46)	6 (24)
** Mixed race**	25 (100)	25 (89)	21 (54)	18 (72)
** Caucasian**	0 (0)	0 (0)	0 (0)	1 (4)

### Performance of Blood Transcriptomic TB Signatures in Pre-Adolescent Children and Adults

**Figure 1:**
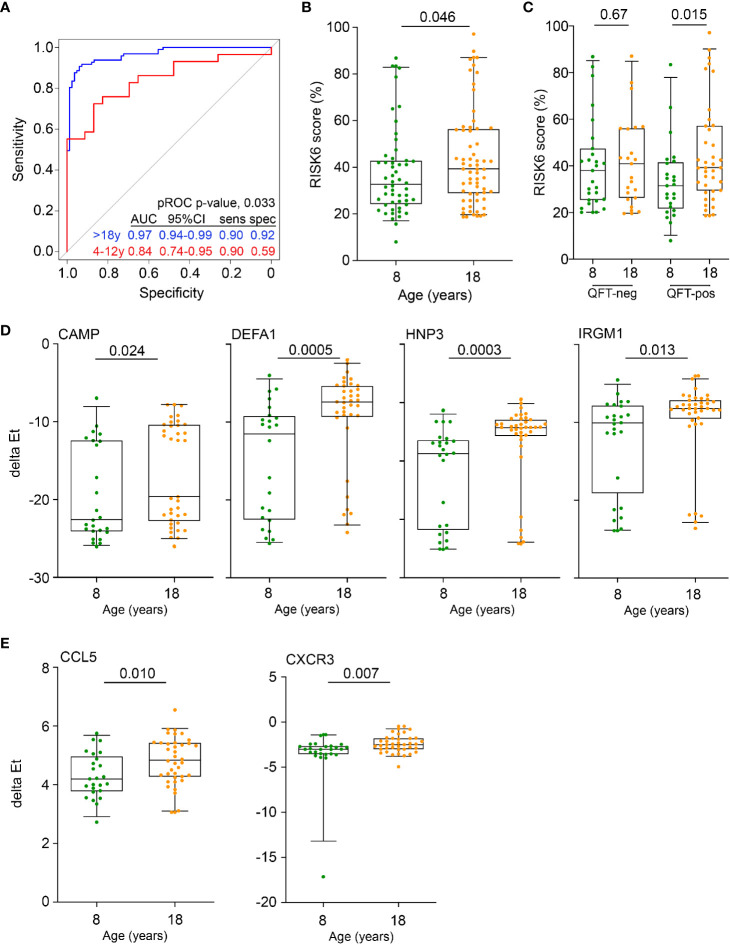
Inflammation and antimicrobial mediators are more abundant in adolescents and adults than in pre-adolescent children. **(A)** Diagnostic performance of the Sweeney3 transcriptomic signature, by analysis of published whole blood microarray data, in differentiating between culture-confirmed TB and LTBI controls in adults (blue) and pre-adolescent children (red). Specificities (spec) are reported at the corresponding sensitivities (sens) of 0.90. The p value for comparing ROC curves in adults and pre-adolescent children was computed using pROC. **(B)** Comparison of RISK6 signature scores, measured by RT-qPCR, between 8- and 18-year-old participants. **(C)** RISK6 signature scores, measured by RT-qPCR, in 8- and 18-year-old participants stratified by QFT status. P values <0.05 were considered significant. **(D)** Expression levels of anti-mycobacterial genes, measured by RT-qPCR, in 8- and 18-year-old QFT+ participants. P values <0.0024 (after adjustment for 21 comparisons using the Bonferroni method) were considered statistically significant. **(E)** Expression levels of myeloid inflammatory genes in 8- and 18-year-old QFT+ participants. P values <0.0023 (after adjustment for 22 comparisons using the Bonferroni method) were considered statistically significant. Horizontal lines depict the median, boxes the interquartile range and whiskers the 95^th^ percentiles. P values were computed with the Mann-Whitney U test.

We first sought to compare the diagnostic performance of Sweeney3, a concise and well-validated blood transcriptomic signature of TB disease (41), that was shown to correlate with the severity of lung inflammation in TB patients (42), in pre-adolescent children and adults. Re-analysis of public whole blood microarray datasets from culture-confirmed TB cases and asymptomatic *M.tb*-infected controls showed that discrimination between TB and controls by Sweeney3 was significantly poorer in pre-adolescent children (AUC 0.84, 95%CI 0.74–0.95), than in adults (AUC 0.97, 95%CI 0.94–0.99, p = 0.03, **Figure 1A**). These data suggest that pre-adolescent children with TB may present with less pronounced inflammation than adult TB cases and motivated further investigation of inflammation as a determinant of age-associated differential risk for TB in healthy individuals. We therefore determined whether signature scores of a recently described parsimonious blood transcriptomic signature of TB risk, RISK6 (28), were different in healthy pre-adolescent vs young adults. We previously showed that RISK6 scores were associated with *in vivo* pulmonary inflammation measured by ^18^F-labeled fluorodeoxyglucose (^18^F FDG) PET-CT in TB patients (28). RISK6 scores, measured by microfluidic RT-qPCR, were significantly higher in 18-year-old young adults than in 8-year-old children (**Figure 1B**). This difference seemed to be primarily driven by persons with underlying *M.tb* infection, since RISK6 scores were only significantly higher in QFT+ adults than in QFT+ children; no difference was observed in the corresponding QFT- groups (**Figure 1C**).

### Expression of Neutrophil Antimicrobial Effectors in Pre-Adolescent Children and Adults

Higher expression of RISK6 in QFT+ adults prompted us to investigate if other mediators of inflammation or antimicrobial responses with a known role in immunity to *M.tb* may also be elevated in this group; we therefore quantified transcript expression by microfluidic RT-qPCR in PBMC from the different participant groups. mRNA expression of the antimicrobial mediators, cathelicidin (CAMP), defensin A1 (DEFA1), human neutrophil peptide 3 (HNP3) and the IFN-inducible immunity-related p47 GTPase IRGM1, were significantly elevated in QFT+ young adults, compared with QFT+ children (**Figure 1D**). A similar picture was observed for the chemokine, CCL5 (encoding RANTES) and the chemokine receptor, CXCR3 (**Figure 1E**). We also compared mRNA expression of these and other genes in QFT- young adults and children, but did not observe such differences (**Supplementary Figure 1**).

### Elevated Myeloid Inflammation in Adults Relative to Pre-Adolescent Children

**Figure 2:**
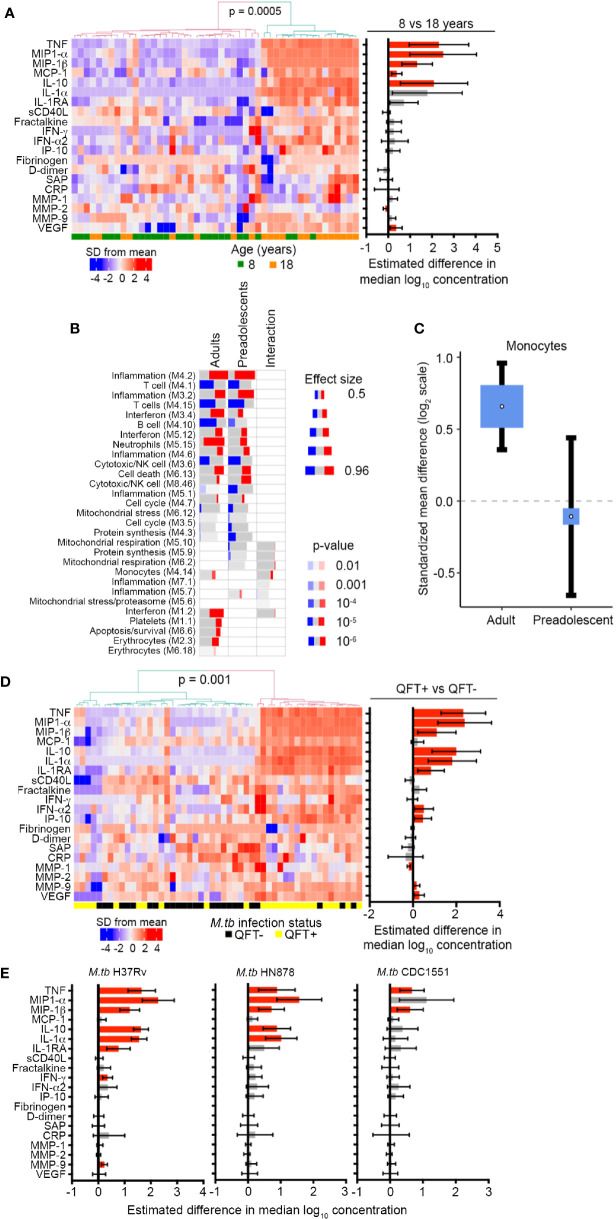
Inflammation and pro-inflammatory, myeloid mediators are more abundant in adolescents and adults than pre-adolescent children and are induced by *in vivo* and *in vitro M.tb* infection. **(A)** Heatmap depicting concentrations of host-derived soluble inflammatory markers in unstimulated blood in QFT- 8- and 18-year-old individuals. The bar graph on the right represents estimated median differences in concentrations of host-derived soluble inflammatory markers between QFT- 8- and 18-year-old individuals. Error bars depict 95% confidence intervals. P values were adjusted using the Benjamini-Hochberg method (by controlling the false discovery rate at 5%) (43). **(B)** Analysis of gene modules, performed using gene set enrichment analysis (GSEA), differentially expressed between TB cases and LTBI controls in adults or pre-adolescent children, as well as the age-associated interaction term between the differentially expressed genes within each module. P values were adjusted using the Benjamini-Hochberg method (by controlling the false discovery rate at 1%). **(C)** Forest plot depicting differences in monocyte abundance between TB cases and LTBI controls in adults or pre-adolescent children, estimated from whole blood gene expression using cell mixture deconvolution. Positive and negative effect sizes indicate higher and lower levels of monocytes in TB versus healthy LTBI controls, respectively. The y axis represents standardized mean difference between TB and LTBI, computed as Hedges’ g, on a log_2_ scale. The size of the blue rectangles is proportional to the SEM difference in the study. Whiskers represent the 95% confidence interval. The white point represents the magnitude of the effect size. **(D)** Heatmap depicting concentrations of host-derived soluble inflammatory markers in unstimulated blood in QFT+ 8-year-old children relative to QFT- 8-year-old children. The bar graph on the right represents estimated median differences in concentrations of host-derived soluble inflammatory markers. **(E)** Estimated median differences and 95% CI (error bars) in concentrations of host-derived soluble inflammatory markers in whole blood from QFT- 8-year-old children in response to *in vitro* stimulation with different strains of live *M.tb*, relative to unstimulated blood. P values were adjusted using the Benjamini-Hochberg method (by controlling the false discovery rate at 5%).

To determine if the inflammatory signals observed by blood mRNA expression profiling were also detectable at the protein level, we measured concentrations of myeloid inflammatory mediators, acute phase proteins and matrix metalloproteinases in plasma from unstimulated blood by Luminex assay. Unsupervised hierarchical clustering of plasma samples based on levels of 20 soluble markers revealed two major sample clusters. The cluster with higher expression of many markers, relative to the other cluster, was significantly enriched for adult samples (Fisher exact, p = 0.0005, **Figure 2A**). When analyzed on a univariate level, concentrations of the myeloid inflammatory mediators, TNF, MIP-1α, MIP-1β, MCP-1, and IL-10, as well as the angiogenic factor, vascular endothelial growth factor (VEGF), were significantly elevated in plasma from QFT- 18-year-old young adults compared with QFT- 8-year-old children (**Figure 2A**). By contrast, MMP-2 was lower whereas IFN-γ, IFN-α2, acute phase proteins D-dimer, serum amyloid protein (SAP) and C-reactive protein (CRP) and the matrix metalloproteinases MMP-1 and MMP-9 were not different. A similar assessment performed for QFT+ 18-year-old adults compared with QFT+ 8-year-old children revealed far fewer differences (**Supplementary Figure 2A**). In light of observing elevated myeloid inflammatory mediators in QFT- adults, we again turned to the public microarray datasets from TB cases and asymptomatic *M.tb*-infected controls to assess whether differential expression of gene modules associated with TB may reveal further biological differences between 4–12-year-old pre-adolescent children and adults. As previously reported (44), many gene modules were differentially expressed between TB cases and LTBI controls, including elevated expression of inflammation, interferon response and myeloid cell subset modules, and lower expression of lymphoid cell (T, B and NK) subset modules in TB (**Figure 2B**). By analyzing age as an interaction term, we detected age-associated differences within the TB vs control comparison. Genes within the mitochondrial respiration, monocyte and interferon modules were more elevated in adults than in pre-adolescent children (**Figure 2B**), again suggesting that myeloid inflammatory mediators were more pronounced in adult TB than in pediatric TB. This difference in the myeloid cell subset was confirmed with the recently published immunoStates computational approach (30) to infer leukocyte representations from gene expression profiles, which also showed that monocytes were significantly elevated in TB cases relative to LTBI controls in adults, but not in pre-adolescent children (**Figure 2C**).

### *In Vitro* and *In Vivo Mycobacterium tuberculosis* Infection Induces Myeloid Inflammation

To address if the observed patterns of age-associated myeloid inflammatory responses can be induced by *in vivo M.tb* infection, we compared plasma levels of inflammatory proteins in QFT+ and QFT- 8-year-old children. Unsupervised hierarchical clustering based on the 20 soluble markers investigated in **Figure 2A** showed that samples segregated into two clusters with higher expression of many markers in the cluster significantly enriched for QFT+ individuals (Fisher exact, p = 0.001, **Figure 2D**). On univariate analysis, myeloid inflammatory mediators including TNF, MIP-1α, MIP-1β, IL-10, IL-1α, IL-1RA, IFN-α2, and IP-10 were significantly higher in QFT+ 8-year-old children than in QFT- 8-year-old children (**Figure 2D**), suggesting that *in vivo* infection with *M.tb*, detected by QFT assay, can induce myeloid inflammatory responses. MMP-9 and VEGF were also higher in QFT+ 8-year-old children, while MMP-1 was lower (**Figure 2D**). However, no differences in soluble marker levels were observed between QFT+ and QFT- 18-year-old young adults (**Supplementary Figure 2B**). To confirm whether *M.tb* can directly induce expression of these inflammatory mediators in blood leukocytes, we analyzed the same markers after *in vitro* stimulation of whole blood with 3 different strains of live *M.tb.* In QFT- 8-year-old children, soluble levels of TNF, MIP-1α, MIP-1β, IL-10, and IL-1α were higher after stimulation of blood with H37Rv and HN878; whereas H37Rv, but not HN878, also induced IL-1RA, IFN-γ and MMP-9 secretion (**Figure 2E**). Stimulation with the *M.tb* strain CDC1551, only induced significant increases in TNF and MIP-1β (**Figure 2E**).

### mRNA Transcripts That Are Higher in 8-Year-Old Children

**Figure 3:**
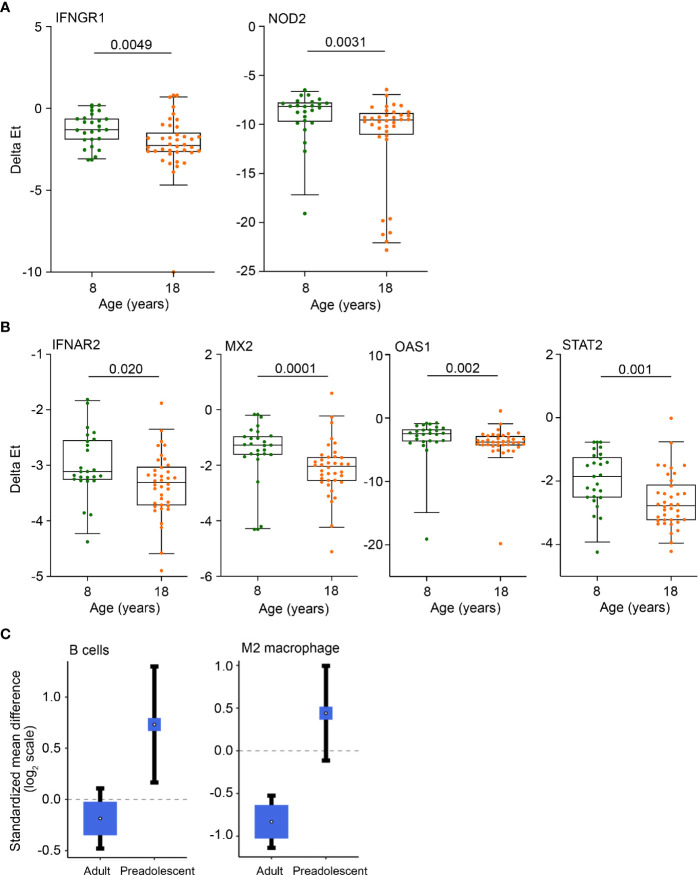
Anti-mycobacterial response and Type I IFN genes, B cells and M2 macrophages are more abundant in pre-adolescent children than adults. **(A, B)** Expression levels of anti-mycobacterial response genes **(A)** and Type 1 IFN response genes **(B)**, measured by RT-qPCR, in 8- and 18-year-old QFT+ participants. Horizontal lines depict the median, boxes the interquartile range and whiskers the 95^th^ percentiles. P values were computed with the Mann-Whitney U test. For **(A)**, p values <0.0024 (after adjustment for 21 comparisons using the Bonferroni method), and for **(B)**, p values <0.0045 (11 comparisons) were considered statistically significant. **(C)** Forest plots depicting differences in B cell and M2 macrophage abundance between TB cases and LTBI controls in adults or pre-adolescent children, estimated from whole blood gene expression using cell mixture deconvolution. Positive and negative effect sizes indicate higher and lower levels of that cell type in TB versus healthy LTBI controls, respectively. The y axes represent standardized mean differences between TB and LTBI, computed as Hedges’ g, on a log_2_ scale. The size of the blue rectangles is proportional to the SEM difference in the study. Whiskers represent the 95% confidence interval. The white point represents the magnitude of the effect size.

These results show that healthy young adults have greater myeloid inflammatory responses than healthy, pre-adolescent children and that monocytes, Type I IFN responses and mitochondrial respiration were more elevated in adult TB than in pediatric TB. We also detected elevated expression of a number of genes in blood from pre-adolescent children relative to young adults, including the IFNGR1 and intracellular pattern recognition receptor, NOD2 (**Figure 3A**) and the Type I IFN stimulated genes IFNAR2, MX2, OAS1 as well as STAT2 (**Figure 3B**). Cellular deconvolution of whole blood gene expression by immunoStates also showed that pre-adolescent children with TB had elevated proportions of B cells and macrophages of the M2 phenotype than LTBI controls, whereas in adults, this difference in B cells was not observed and M2 macrophages were significantly lower in TB cases (**Figure 3C**).

## Discussion

The risk of progression from *Mycobacterium tuberculosis* infection to active disease is significantly lower in pre-adolescent children above 4 years of age than in post-pubescent adolescents and young adults (3–5). Further, the typical clinical presentation of TB manifests as mild and/or pauci-bacillary lymph node disease in pre-adolescent children, whereas post-pubescent adolescents and adults more commonly present with multi-bacillary, “adult-type” pulmonary disease (3–5). To investigate possible immunological underpinnings of these age-associated characteristics of TB, we compared blood gene expression of antimicrobial effector molecules, pro- and anti-inflammatory and other immune mediators as well as soluble host-derived inflammatory molecules in pre-adolescent children and young adults.

In light of the well-described links between excessive innate inflammation and risk of TB progression as well as the role of inflammation as a cause of immunopathology and tissue destruction (12, 45, 46), we hypothesized that pro-inflammatory responses to *M.tb* in pre-adolescent children are either less pronounced or more regulated, than in young adults.

Three main findings emerged from our analyses: 1) diagnostic performance of transcriptomic signatures of TB for differentiating between culture-confirmed TB and LTBI controls was poorer in pre-adolescent children than in adults. 2) Pre-adolescent children had lower levels of myeloid-associated pro-inflammatory mediators than young adults. These mediators were induced by *in vivo* and *in vitro M.tb* infection, as supported by higher expression in QFT+ than QFT- 8-year-old children and significantly higher expression after *in vitro* stimulation of blood with live *M.tb*. 3) Compared with young adults, pre-adolescent children had higher levels of IFN stimulated genes IFNAR2, MX2, OAS1 and STAT2, as well as B cells and M2 macrophages than adults.

The “Golden Age” associated poorer performance of the previously published Sweeney3 signature of TB in differentiating between TB and LTBI controls, the lower RISK6 signature scores and the lower levels of myeloid-associated pro-inflammatory mediators all support our hypothesis that children have lower inflammation than young adults. Importantly, we observed this difference in both healthy individuals and in those with TB disease, suggesting that this finding did not only reflect differences in TB disease manifestation, but that these age-associated outcomes may reflect intrinsic differences that are not only restricted to mycobacterial immune responses. This is also supported by the results from our previous study of mycobacteria-specific T cell responses and mycobacterial growth inhibition in the same 8- and 18-year-old cohort; no age-associated differences in these common measures of mycobacteria-associated immune outcomes were observed (7). The observation that M2 macrophages, which are typically anti-inflammatory, were more abundant in pre-adolescent children than adults, further supports this. We were not able to definitively interpret the higher abundance of B cells in pre-adolescent children, since this designation includes many subclasses of B cells with different functions. Ultimately, this finding may simply reflect greater lymphocyte proportions in pre-adolescent children than adults.

Our observations are consistent with the hypothesis that pro- and anti-inflammatory responses must be balanced to avoid bias towards an excessive response (8, 9) and suggest that pre-adolescent children may be more likely to maintain an optimal balance. If this is true, then similar age-associated differences must exist for other infections and diseases, including autoimmune and inflammatory conditions. Indeed, sarcoidosis, a granulomatous disease associated with dysregulated IFN responses (47, 48), is not commonly reported in young, pre-adolescent children and increases after puberty (49). Among other infectious diseases, cutaneous leishmaniasis, schistosomiasis and leptospirosis follow the same curve that is observed for TB (50), with lowest incidences in pre-adolescent children above 4 years of age. While many factors affect the risk for these diseases, as is also true for TB, we hypothesize that the age-associated balance of inflammatory responses plays a role.

It was interesting that we observed higher expression of multiple Type I IFN mRNAs in pre-adolescent children compared with young adults. Our integrated analyses of differences in global gene expression between *M.tb* infection and TB disease, also revealed modest, but interesting age-associated differences in transcriptional programs typically associated with human TB, namely interferon signaling and myeloid inflammation (51, 52). To our knowledge, no study has hitherto teased apart the transcriptional program within each age stratum in the context of the observed epidemiological difference in TB disease incidence and pathophysiology, which we reveal in our analyses. We observed more exacerbated transcriptional dysregulation in interferon signaling in adults when compared to pre-adolescent children. Notably, pathways associated with mitochondrial stress/proteasome and mitochondrial respiration, suggestive of impaired mitochondrial function, which may be linked to necrotizing granulomatous lesions often observed in adult TB disease or, alternatively, may be related to dysregulated inflammatory processes. Mitochondria mass, size, number, and fragmentation is known to be affected by virulent *M.tb* after macrophage infection (53) and recent findings by Pajuelo and colleagues provided evidence that *M.tb* hijacks a host cell death pathway involving the mitochondrion (54). Our results may at first appear to contradict the hypothesized higher inflammation in adults, especially in light of reports that Type I IFN responses predispose to TB disease progression (13, 55, 56). However, the biology of Type I IFN mRNAs is known to be particularly influenced by immunological changes during sexual maturation during and after puberty, which lead to divergence in immune and inflammatory responses between males and females. For example, females have higher expression of Type I IFN upon TLR7 stimulation than males (57), and the effects of oestrogens typically lead to higher levels of inflammation in females, while progesterone and androgens, such as testosterone, generally mediate immunosuppressive activities (58, 59). Since TB notification rates in males are typically double those in females (1), and Type I IFN responses are typically higher in females, we propose that it may be too simplistic to expect a direct association between risk of TB and Type I IFN responses.

We acknowledge that our results reflect peripheral blood and not the site of disease, which limits our ability to directly establish a link between mitochondrial function and events at the site of disease. Our work is also subject to other limitations. The relatively small sample size limited statistical power to definitively address the influence of covariates, such as sex and ethnicity, on immunological outcomes and also did not allow analyses using age as a continuous variable. The restriction of our enrolled cohort of children and young adults to very specific ages (8 and 18 years of age) also limits the generalizability of our conclusions. Furthermore, analysis of the influence of underlying *M.tb* infection on age-associated immunological outcomes is limited by the absence of a true measure of *in vivo M.tb* in humans. It is well known that IFN-γ release assays, including the QFT assay we used to infer *M.tb* infection, are at best imperfect tests for infection (60). Finally, we note that the age-associated immunological differences observed in our study are modest in magnitude. In light of the many well-known risk factors for TB (61), independent replication of our findings is necessary.

In summary, our results suggest that myeloid inflammation is intrinsically lower in pre-pubescent children than in young adults and that this may play a role in age-associated differential risk of TB and/or the differences in clinical presentation of this disease. Further dissection of these age-associated differences may reveal opportunities to develop interventions that aim to optimize the balance between pro- and anti-inflammatory responses.

## Data Availability Statement

New data was generated and analysed in this study and can be found in the following online repository: https://doi.org/10.25375/uct.13351466.v1. Publicly available datasets were also analysed in this study. This data can be found here: Gene Expression Omnibus, https://www.ncbi.nlm.nih.gov/geo/, GSE39940 and GSE37250.

## Ethics Statement

Participants were recruited under protocols approved by the University of Cape Town Human Research Ethics Committee. Written informed consent was obtained from adults prior to enrolment. Children provided written informed assent while their legal guardians provided written informed consent prior to enrollment.

## Author Contributions

MH, APN and TJS designed the study. MdK, MvR, LS and MH performed clinical investigations. RB, ME, JD, LM, MdK, NB, MS, HA, FD and NC processed samples or performed assays. RB, SKM, MR, JD, FD, NC, GT, GW, APN and TJS analysed and interpreted the data. RB, SKM, APN and TJS wrote the manuscript. All authors contributed to the article and approved the submitted version.

## Funding

This work was supported by grants from the European and Developing Countries Clinical Trials Partnership (TA.2011.40200.010) and from the European Commission funded TBVAC2020 Consortium (H2020-PHC-643381). The funders were not involved in the research.

## Conflict of Interest

AP-N and TS have a patent of the RISK6 signature pending.

The remaining authors declare that the research was conducted in the absence of any commercial or financial relationships that could be construed as a potential conflict of interest.

The handling editor declared a past co-authorship with several of the authors, TS and MH.
