# Supplementary material for: Inflammatory Determinants of Differential Tuberculosis Risk in Pre-Adolescent Children and Young Adults
Source: Front Immunol. 2021 Feb 25;12:639965. doi: 10.3389/fimmu.2021.639965 (PMC7947716; doi:10.3389/fimmu.2021.639965)
Supplement: Supplementary file 1 [file DataSheet_1.pdf]

Table S1. Microfluidic panel gene list with 87 genes of interest and 9 housekeeping (reference) genes

Gene Symbol	TaqMan Assay ID	Function	Gene Symbol	TaqMan Assay ID	Function	Gene Symbol	TaqMan Assay ID	Function
ALOX5	Hs01095330_m1	Inflammation	MMP9	Hs00957562_m1	Immunopathology	IRF8	Hs01128710_m1	IFN I response
ALOX12	Hs00167524_m1	Inflammation	TIMP1	Hs9999139_m1	Immunopathology	MX1	Hs00895608_m1	IFN I response
CCL5	Hs00174575_m1	Inflammation	TIMP2	Hs00234278_m1	Immunopathology	MX2	Hs01550811_m1	IFN I response
CXCL10	Hs00171042_m1	Inflammation	BPI	Hs01552756_m1	Antimycobacterial	OAS1	Hs00973637_m1	IFN I response
CXCR3	Hs01847760_s1	Inflammation	CAMP	Hs00189038_m1	Antimycobacterial	ISG15	Hs01921425_s1	IFN I response
FCGR2A	Hs01017702_g1	Inflammation	DEFA1	Hs00234383_m1	Antimycobacterial	CD274	Hs01125299_m1	Regulatory
GMCSF	Hs00929873_m1	Inflammation	DEFA4	Hs01056651_g1	Antimycobacterial	CTLA4	Hs03044418_m1	Regulatory
IL1B	Hs01555410_m1	Inflammation	GNLY	Hs00246266_m1	Antimycobacterial	FOXP3	Hs01085834_m1	Regulatory
IL12A	Hs00168405_m1	Inflammation	GZMA	Hs00989184_m1	Antimycobacterial	GARP	Hs01017468_m1	Regulatory
IL12B	Hs01011519_m1	Inflammation	GZMB	Hs01554355_m1	Antimycobacterial	IL10	Hs00961622_m1	Regulatory
IL22	Hs01574154_m1	Inflammation	HNP3	Hs00414018_m1	Antimycobacterial	STAT2	Hs01013123_m1	Regulatory
IL32	Hs00992441_m1	Inflammation	IFNG	Hs00989291_m1	Antimycobacterial	TGFB	Hs00998133_m1	Regulatory
IL6	Hs00985639_m1	Inflammation	IFNGR1	Hs00166223_m1	Antimycobacterial	SOCS1	Hs00705164_s1	Regulatory
IL8	Hs99999034_m1	Inflammation	IL15	Hs01003716_m1	Antimycobacterial	GBP2	Hs00894846_g1	RISK6
IL1RA	Hs00893626_m1	Inflammation	IL15RA	Hs00233692_m1	Antimycobacterial	FCGR1B	Hs02341825_m1	RISK6
LTA4H	Hs01075871_m1	Inflammation	IL27	Hs00377366_m1	Antimycobacterial	SERPING1	Hs00934329_m1	RISK6
MCP1	Hs00234140_m1	Inflammation	LTF	Hs00914334_m1	Antimycobacterial	SDR39UI	Hs01016970_g1	RISK6
MCP2	Hs04187715_m1	Inflammation	MARCO	Hs00198935_m1	Antimycobacterial	TUBGCP6	Hs00363509_g1	RISK6
MIP1B	Hs01031494_m1	Inflammation	NOD2	Hs00223394_m1	Antimycobacterial	TRMT2A	Hs01000041_g1	RISK6
TLR8	Hs00607866_mH	Inflammation	PRF1	Hs00169473_m1	Antimycobacterial	ACTR3	Hs01029159_g1	Reference
TNFA	Hs01113624_g1	Inflammation	SLAMF7	Hs00221793_m1	Antimycobacterial	ADRBK1	Hs01056343_g1	Reference
TNFR1	Hs01042313_m1	Inflammation	STAB1	Hs01109068_m1	Antimycobacterial	CDC42	Hs03044122_g1	Reference
TNFR2	Hs00961748_m1	Inflammation	CYPD	Hs04193937_gH	Antimycobacterial	CSDE1	Hs00918650_m1	Reference
TNFRSF25	Hs00237056_m1	Inflammation	IRGM1	Hs01013699_s1	Antimycobacterial	CYTIP	Hs00188734_m1	Reference
CASP1	Hs00354836_m1	Immunopathology	PTGES2	Hs00228159_m1	Antimycobacterial	TMBIM6	Hs01012081_m1	Reference
CASP3	Hs00234387_m1	Immunopathology	VDR	Hs00172113_m1	Antimycobacterial	TMBIM6	Hs01012082_g1	Reference
CASP5	Hs00362078_m1	Immunopathology	DDX58	Hs00204833_m1	IFN I response	TPM3	Hs01900726_g1	Reference
MMP1	Hs00899658_m1	Immunopathology	IFI16	Hs00194261_m1	IFN I response	USF2	Hs01100994_g1	Reference
MMP14	Hs01037009_g1	Immunopathology	IFNAR	Hs01066118_m1	IFN I response	PTGER2	Hs04183523_m1	Other
MMP2	Hs01548727_m1	Immunopathology	IFNAR2	Hs01022060_m1	IFN I response	RAB33A	Hs00191243_m1	Other
MMP25	Hs01554789_m1	Immunopathology	IRF1	Hs00971960_m1	IFN I response	TREM1	Hs00218624_m1	Other
MMP8	Hs01029057_m1	Immunopathology	IRF7	Hs01014809_g1	IFN I response	RPL13A	Hs04194366_g1	Other

Table S2. Summary of publicly available microarray datasets used for comparisons among the latent TB infection and active TB disease.

GSE	Age group (years)	Disease status	
		LTBI	Active TB
37250	> 18	83	97
39940	4 - 12	23	29

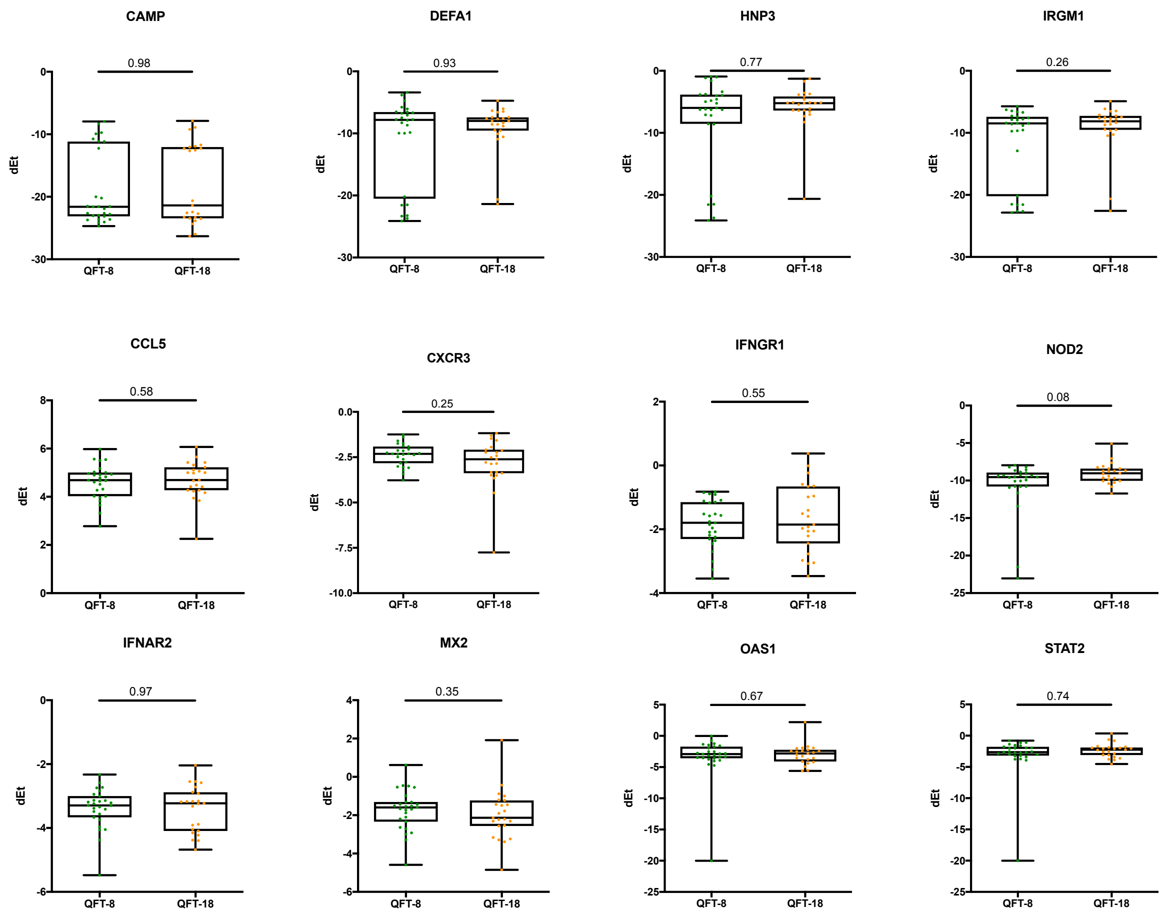

Supplementary Figure 1. mRNA transcript expression in uninfected (QFT-) pre-adolescent children and young adults. Expression levels of anti-mycobacterial response genes, myeloid inflammation genes and Type 1 IFN response genes, measured by RT-qPCR, in 8 and 18 year old QFT- participants. Horizontal lines depict the median, boxes the interquartile range and whiskers the 95th percentiles. P values were computed with the Mann-Whitney U test.

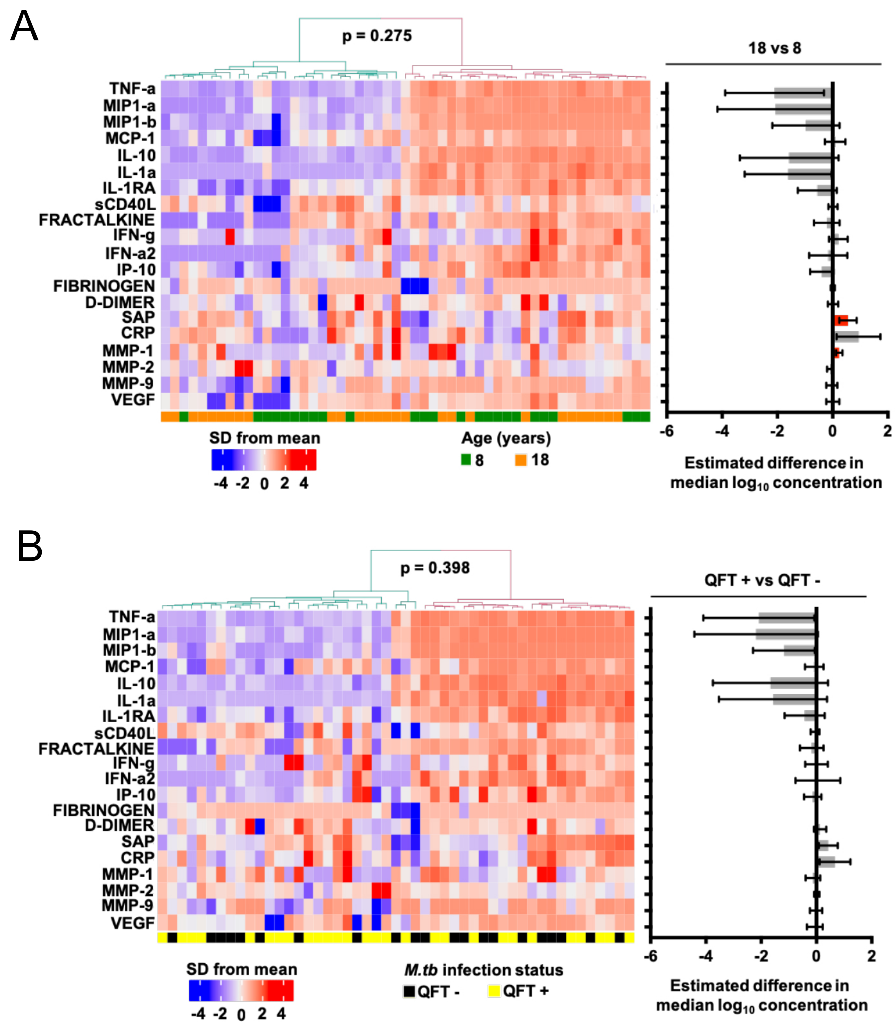

Supplementary Figure 2. Inflammation and pro-inflammatory, myeloid mediators in QFT+ pre-adolescent children and young adults. (**A**) Heatmap depicting concentrations of host-derived soluble inflammatory markers in unstimulated blood from QFT+ 8 and QFT+ 18 year old individuals. (**B**) Heatmap depicting concentrations of host-derived soluble inflammatory markers in unstimulated blood from QFT+ and QFT- 18 year old individuals. The bar graphs on the right represent estimated median differences in concentrations of host-derived soluble inflammatory markers between the groups compared on the left. Error bars depict 95% confidence intervals.
